# Corneal transplantation for keratoconus in South Korea

**DOI:** 10.1038/s41598-021-92133-y

**Published:** 2021-06-15

**Authors:** Sungsoon Hwang, Tae-Young Chung, Jisang Han, Kyunga Kim, Dong Hui Lim

**Affiliations:** 1 Department of Ophthalmology, Samsung Medical Center, Sungkyunkwan University School of Medicine, #81 Irwon-ro, Gangnam-gu, Seoul, 06351 Republic of Korea; 2 Department of Clinical Research Design and Evaluation, Samsung Advanced Institute for Health Sciences and Technology (SAIHST), Sungkyunkwan University, Seoul, Republic of Korea; 3 Department of Ophthalmology, Kangbuk Samsung Hospital, Sungkyunkwan University School of Medicine, Seoul, Republic of Korea; 4 Statistics and Data Center, Research Institute for Future Medicine, Samsung Medical Center, Seoul, Republic of Korea; 5 Department of Digital Health, Samsung Advanced Institute for Health Sciences and Technology (SAIHST), Sungkyunkwan University, Seoul, Republic of Korea

**Keywords:** Corneal diseases, Epidemiology

## Abstract

This nationwide population-based study investigated the incidence rate of and risk factors for the progression to corneal transplantation in patients with keratoconus in South Korea using claims data from the Health Insurance Review and Assessment service. Among the entire South Korean population, 10,612 patients newly diagnosed with keratoconus between January 2010 and June 2015 were identified and included in the study. During the study period, 124 patients (1.17%) underwent corneal transplantation, with an average follow-up period of 2.97 ± 1.59 years. The incidence rate of corneal transplantation in patients with keratoconus was 4.46 cases per 1000 person-years. The rate of corneal transplantation for keratoconus was relatively low in South Korea compared to other countries. Multivariate Cox regression analysis revealed that male sex (HR 2.37; 95% CI 1.61–3.50; P < 0.001), severe atopic dermatitis (HR 2.32; 95% CI 1.02–5.28; P = 0.044), obstructive sleep apnea (HR 9.78; 95% CI 1.36–70.10; P = 0.023), and intellectual disability (HR 4.48; 95% CI 1.33–15.11; P = 0.016) significantly increased the risk of progression to corneal transplantation. In patients with keratoconus, male sex, severe atopic dermatitis, obstructive sleep apnea, and intellectual disability were associated with an increased risk of corneal transplantation.

## Introduction

Keratoconus is a chronic, non-inflammatory, and progressive corneal disease characterized by corneal stromal thinning, resulting in irregular astigmatism and visual deterioration^1,2^. Keratoconus normally affects people in their second to fourth decade of life^3^, and can clinically range from a mild subclinical form (or forme fruste) to a severe progressive form, which may include corneal scarring, hydrops, perforation, or blindness^3,4^.

There are several treatment options for keratoconus. Some cases of keratoconus can be managed with glasses or contact lenses. Corneal interventions, such as intrastromal corneal ring segments^5,6^ and riboflavin/ultraviolet A induced corneal collagen cross-linking^7–9^, can stabilize disease progression. However, approximately 3–20% of patients with keratoconus require corneal transplantation when the keratoconus is severe at presentation or continues to worsen despite intervention^2,4,10–13^.

Keratoconus is a multifactorial disease caused by a combination of genetic and environmental factors^14^. Demographic characteristics, including ethnicity, age, sex, socio-economic status, and certain comorbidities such as atopy, connective tissue disease, diabetes, obstructive sleep apnea, and Down syndrome, are also reportedly associated with keratoconus^15,16^. However, few studies have assessed the factors associated with progression to corneal transplantation in patients with keratoconus^13,17^.

Therefore, in the present study, we investigated the incidence of and risk factors associated with corneal transplantation in patients with keratoconus in South Korea.

## Methods

### Setting

This is a nationwide population-based retrospective cohort study. We used data from the Korean National Health claims database of patients with keratoconus. The investigators accessed health claim records from January 2009 to June 2015 provided by the Health Insurance Review and Assessment (HIRA) service of South Korea. The HIRA service reviews all health claims made in South Korea submitted through the Korean National Health Insurance scheme, which covers 97% of the South Korean population, and through other available medical assistance programs (such as the Medical Assistance Program and Medical Care for Patriots and Veterans Affairs Scheme), which cover the remaining 3% of the population. The HIRA database stores data on the diagnoses, visit dates, procedures, prescription records, comorbidities, and demographic information of the entire South Korean population. This database has been used widely, including in several studies that have identified novel determinants of various diseases. Details regarding the HIRA database have been provided in previous studies^18,19^.

The HIRA Deliberative Committee approved the use of data collected from January 2009 to June 2015 in the HIRA database based on the understanding that all identifiable personal information would be de-identified. The study adhered to the principles of the Declaration of Helsinki and was approved by the Samsung Medical Center Institutional Review Board/Ethics Committee (SMC2016-04-062). The board waived the requirement for informed consent based on the use of de-identified public data and the retrospective nature of the study.

### Participants

From among the entire South Korean population, patients who were diagnosed with keratoconus (registration code H18.6) during the study period were initially included. Additionally, patients with keratoconus diagnostic codes (medical specialty code “12”) that were registered at ophthalmology clinics or departments were included.

**Figure 1:**
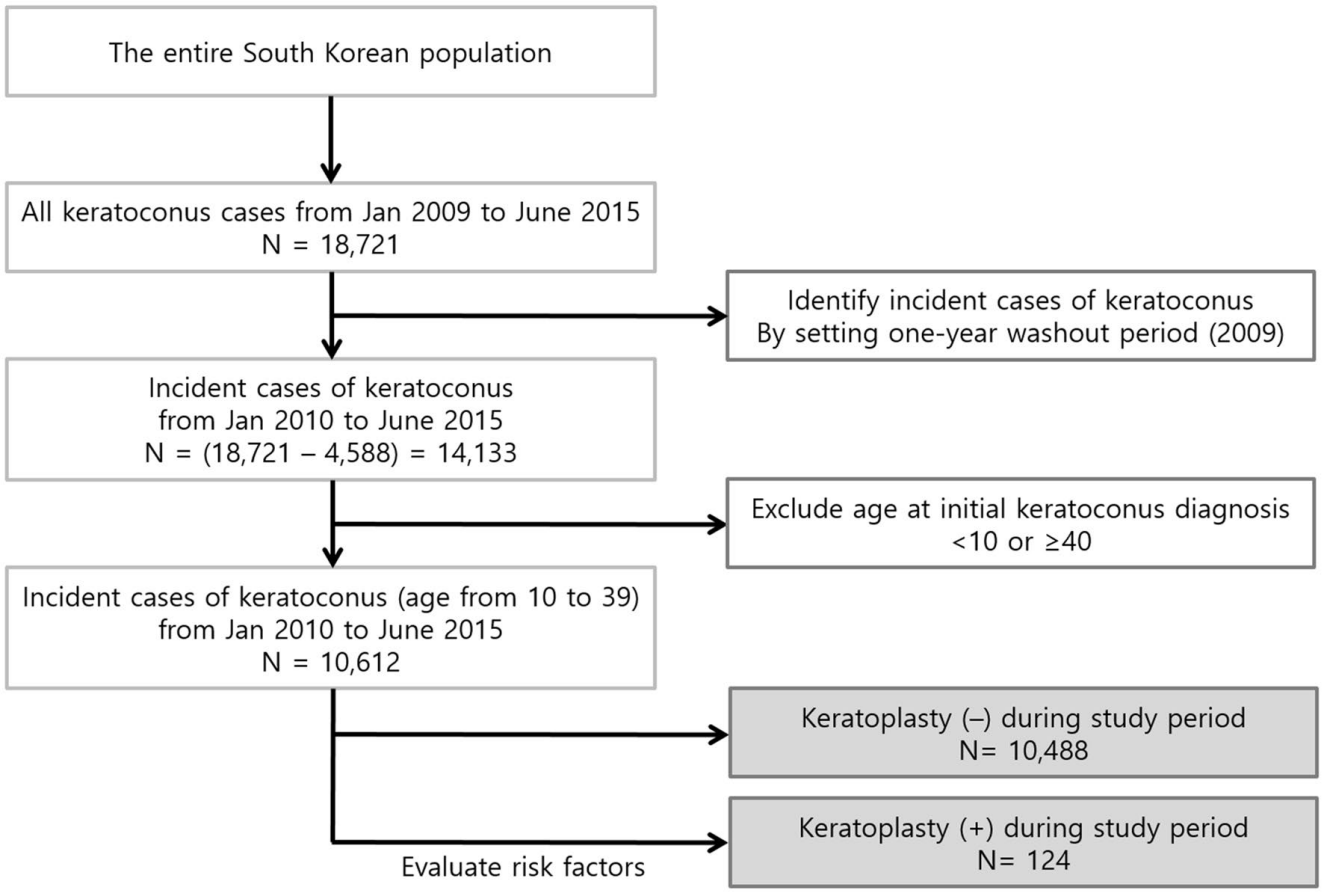
Flowchart for the identification of patients with incident keratoconus and the progression to corneal transplantation.

To identify incident cases of keratoconus, the investigator established a 1-year washout period and excluded cases identified in the first year (2009) to eliminate any potential pre-existing cases of keratoconus before 2009^18^. The date of the earliest claim with a registration code for keratoconus was defined as the date of initial diagnosis. Patients aged < 10 years and ≥ 40 years at their initial diagnoses were excluded because most cases of keratoconus during this period are either inactive or progress significantly. Among patients with incident keratoconus from January 2010 to June 2015, those who underwent penetrating keratoplasty (registration code S5372) or anterior lamellar keratoplasty (registration code S5371) were identified. The process for the identification of patients with incident keratoconus and subsequent corneal transplantation is shown in Fig. 1. According to statistics from the Korean National Health Insurance Service, only 1% of those enrolled were foreigners (https://nhiss.nhis.or.kr/bd/ad/bdada024cv.do, accessed December 31, 2020); therefore, most patients included in this study were Korean^18^.

### Defining the risk factors

The risk factors included in the statistical analysis were sex, age, and income level at the initial diagnosis of keratoconus, and pre-existing systemic diseases (such as hypertension, diabetes mellitus, dyslipidemia, atopic dermatitis, asthma, allergic rhinitis, collagen vascular disease, aortic aneurysm, mitral valve prolapse, and obstructive sleep apnea) or congenital diseases (intellectual disability, Down syndrome, Turner syndrome, Marfan syndrome, Ehlers–Danlos syndrome, and osteogenesis imperfecta). Income levels were categorized into quartiles according to the insurance premium level, which was based on the household income. A pre-existing systemic disease was defined as the diagnosis of a disease within 1 year of the initial diagnosis of keratoconus. We assumed that people would visit clinics at least once a year if they had certain diseases that required treatment or regular follow-up. Patients with diagnostic codes for congenital diseases at any time during the study period were considered to have pre-existing congenital diseases. Severe atopic dermatitis was defined based on the prescription of drugs according to established guidelinees^20,21^. Specifically, patients were considered to have severe atopic dermatitis if they were undergoing rituximab, omalizumab, immunoglobulin, or interferon-γ and/or cyclosporine, azathioprine, mycophenolate mofetil, methotrexate, or systemic steroid treatment for 2 or more weeks under the atopic dermatitis diagnostic code in the same claim. Details regarding the diagnostic and medication codes used for defining the presence of pre-existing diseases are listed in Supplementary Table S1.

### Statistical analysis

The patients were divided into two groups based on whether they had undergone corneal transplantation during the study period. The baseline characteristics were compared between the two groups using the independent *t* test for continuous variables and the Fisher’s exact test for categorical variables. The incidence of corneal transplantation after the initial diagnosis of keratoconus per person-years was also calculated. To evaluate the hazard ratio (HR) and 95% confidence interval (CI) for the risk factors for corneal transplantation, univariate and multivariate Cox regression analyses were conducted. An event was defined as the first corneal transplantation after the diagnosis of keratoconus, and the time period was defined as the period between the initial diagnosis of keratoconus and the first corneal transplantation or the period between the initial diagnosis of keratoconus and the last follow-up date. Parameters with P values < 0.1 in the univariate analysis were included in the subsequent multivariate analysis. P values < 0.05 were considered statistically significant. All statistical analyses were performed using the Statistical Analysis System software version 9.4 (SAS Inc., Cary, NC, USA).

## Results

Among the entire South Korean population, 18,721 cases of keratoconus were identified from January 2009 to June 2015. After applying the 1-year washout period and setting the age limit for active keratoconus, 10,612 cases of incident keratoconus were finally included in the study (Fig. 1).

A total of 124 patients (1.17%) underwent corneal transplantation during an average follow-up period of 2.97 ± 1.59 years. The time from the initial diagnosis of keratoconus to the first corneal transplantation was 1.25 ± 1.19 years. Among the 124 cases, 22 underwent anterior lamellar keratoplasty and 102 underwent penetrating keratoplasty.

Table 1 shows the baseline characteristics and the distribution of risk factors for corneal transplantation in patients with keratoconus. The proportion of patients that were men and that had severe atopic dermatitis, obstructive sleep apnea, intellectual disability, and Down syndrome was higher among those who underwent corneal transplantation, while the proportion of patients with diabetes mellitus was lower.

**Table 1:** Descriptive characteristics of patients with keratoconus who did and did not undergo corneal transplantation.

	Corneal transplantation		P value*
	No (n = 10,488)	Yes (n = 124)	
**Age group (years)**	26.22 ± 6.83	26.01 ± 6.42	0.746
10–19	1987 (18.95%)	25 (20.16%)	0.895
20–29	4926 (46.97%)	56 (45.16%)	
30–39	3575 (34.09%)	43 (34.68%)	
**Sex (male)**			< 0.001
Male	5473 (52.18%)	88 (70.97%)	
Female	5015 (47.82%)	36 (29.03%)	
**Income level**			0.624
Quartile 1 (low income)	2251 (21.46%)	24 (19.35%)	
Quartile 2	2466 (23.51%)	25 (20.16%)	
Quartile 3	2993 (28.54%)	41 (33.06%)	
Quartile 4 (high income)	2778 (26.49%)	34 (27.42%)	
**Baseline comorbidities**			
Hypertension	153 (1.46%)	1 (0.81%)	1.000
Diabetes mellitus	460 (4.39%)	1 (0.81%)	0.045
Dyslipidemia	447 (4.26%)	4 (3.23%)	0.821
Atopic dermatitis	500 (4.77%)	7 (5.65%)	0.668
Atopic dermatitis (severe)	240 (2.29%)	6 (4.84%)	0.069
Asthma	763 (7.27%)	6 (4.84%)	0.383
Allergic rhinitis	4997 (47.64%)	54 (43.55%)	0.368
Collagen vascular disease	40 (0.38%)	1 (0.81%)	0.383
Aortic aneurysm	1 (0.01%)	0 (0.00%)	1.000
Mitral valve prolapse	0 (0.00%)	0 (0.00%)	–
Obstructive sleep apnea	9 (0.09%)	1 (0.81%)	0.111
Intellectual disability	51 (0.49%)	4 (3.23%)	0.004
Down syndrome	17 (0.16%)	2 (1.61%)	0.020
Turner syndrome	0 (0.00%)	0 (0.00%)	–
Marfan syndrome	1 (0.01%)	0 (0.00%)	1.000
Ehlers–Danlos syndrome	0 (0.00%)	0 (0.00%)	–
Osteogenesis imperfecta	1 (0.01%)	0 (0.00%)	1.000

*P values were determined using the independent *t* test for continuous variables and Fisher’s exact test for categorical variables.

Table 2 provides detailed information on the incidence rate of corneal transplantation in patients with keratoconus and the HRs and 95% CIs of the risk factors for progression to corneal transplantation as evaluated using univariate and multivariate Cox regression analyses. The overall incidence rate of corneal transplantation in patients with keratoconus was 4.463 cases per 1000 person-years. The P values for sex, diabetes mellitus, severe atopic dermatitis, obstructive sleep apnea, intellectual disability, and Down syndrome were < 0.1 in the univariate Cox regression analysis. Rare diseases, such as aortic aneurysm, mitral valve prolapse, Turner syndrome, Marfan syndrome, Ehlers–Danlos syndrome, and osteogenesis imperfecta, were not included in the risk factor analysis because there were only a few patients with these diseases. Subsequent multivariate Cox regression analysis revealed that male sex and presence of severe atopic dermatitis, obstructive sleep apnea, and intellectual disability significantly increased the risk of progression to corneal transplantation in keratoconus patients. The HR for diabetes mellitus was low but not statistically significant (P = 0.072).

**Table 2:** Incidence rate and risk factors for progression to corneal transplantation in patient with keratoconus using univariate and multivariate Cox regression analyses.

	Status	Incidence rate*	Univariate		Multivariate	
			HR (95% CI)	P value	HR (95% CI)	P value
Overall		4.463	–	–	–	–
Age group (years)	10–19	4.743	1.000 (reference)	–	–	–
	20–29	4.217	0.896 (0.559–1.436)	0.648	–	–
	30–39	4.658	0.977 (0.597–1.599)	0.925	–	–
Sex	Female	2.630	1.000 (reference)	–	1.000 (reference)	–
	Male	6.243	2.347 (1.592–3.460)	< 0.001	2.370 (1.605–3.497)	< 0.001
Income level	Quartile 1	4.029	1.000 (reference)	–	–	–
	Quartile 2	3.833	0.972 (0.555–1.703)	0.922	–	–
	Quartile 3	5.161	1.304 (0.788–2.157)	0.302	–	–
	Quartile 4	4.618	1.155 (0.685–1.948)	0.588	–	–
Hypertension	No	4.494	1.000 (reference)	–	–	–
	Yes	2.415	0.537 (0.075–3.841)	0.536	–	–
Diabetes mellitus	No	4.647	1.000 (reference)	–	1.000 (ref.)	–
	Yes	0.758	0.167 (0.023–1.196)	0.075	0.164 (0.023–1.175)	0.072
Dyslipidemia	No	4.504	1.000 (reference)	–	–	–
	Yes	3.513	0.773 (0.286–2.094)	0.613	–	–
Atopic dermatitis	No	4.425	1.000 (reference)	–	–	–
	Yes	5.215	1.172 (0.547–2.513)	0.683	–	–
Atopic dermatitis (severe)	No	4.346	1.000 (reference)	–	1.000 (ref.)	–
	Yes	9.503	2.148 (0.946–4.876)	0.068	2.323 (1.022–5.281)	0.044
Asthma	No	4.578	1.000 (reference)	–	–	–
	Yes	2.988	0.645 (0.284–1.465)	0.295	–	–
Allergic rhinitis	No	4.832	1.000 (reference)	–	–	–
	Yes	4.062	0.837 (0.587–1.194)	0.326	–	–
Collagen vascular	No	4.446	1.000 (reference)	–	–	–
	Yes	8.447	1.933 (0.270–13.829)	0.511	–	–
Obstructive sleep apnea	No	4.432	1.000 (reference)	–	1.000 (ref.)	–
	Yes	38.091	8.538 (1.193–61.092)	0.033	9.775 (1.363–70.104)	0.023
Intellectual disability	No	4.341	1.000 (reference)	–	1.000 (ref.)	–
	Yes	28.609	6.674 (2.465–18.071)	< 0.001	4.479 (1.329–15.105)	0.016
Down syndrome	No	4.400	1.000 (reference)	–	1.000 (ref.)	–
	Yes	34.784	8.070 (1.995–32.637)	0.003	3.882 (0.703–21.451)	0.120

*HR* hazard ratio, *CI* confidence interval. *Incidence rate per 1000 person-years.

## Discussion

In the present study, only 1.16% of patients with keratoconus underwent corneal transplantation during the mean follow-up period of 2.97 ± 1.59 years, which is much lower than what has previously been reported. Tuft et al. reported that 21.6% of the 5242 eyes studied underwent corneal transplantation during an average follow-up period of 54 months^10^, and the Collaborative Longitudinal Evaluation of Keratoconus study group reported that the eight-year incidence of corneal transplantation was 15% of the 2418 eyes of participants aged < 40 years^22^. The low rate of corneal transplantation in patients with keratoconus in our study compared to that in previous large-scale studies might be attributable to the following reasons: (1) the follow-up period in our study was shorter than that of previous studies; therefore, we might have missed patients who underwent corneal transplantation after the study period and (2) the previous studies reported were published before imaging modalities had been improved; therefore, our study may comprise a large proportion of patients with mild keratoconus since it is now diagnosed more frequently and much earlier than it was in the past. Furthermore, the patients in previous studies could not undergo corneal procedures that have recently been introduced (e.g., collagen cross-linking) which prevent progression of keratoconus. However, even in a recent study performed in the United States, which had a similar study design to ours, 3.17% (684 out of 21,588) of patients with keratoconus underwent corneal transplantation^13^, which is three times that of the present study. Our team previously reported that, contrary to the notion that keratoconus is more prevalent among Asians, the incidence of keratoconus in the East Asian population was found to be lower than that among other ethnicities^18^. The low incidence of corneal transplantation for keratoconus in South Korea might be explained by the relatively milder type of keratoconus that affects South Koreans compared to the type that affects other populations. However, further studies are needed to validate this finding.

Some studies have already reported the risk factors for corneal transplantation in patients with keratoconus. Reeves et al. conducted a case–control study in a single tertiary medical center and reported that young age, high keratometric value, astigmatism, and worse best-corrected visual acuity are associated with an increased risk of corneal transplantation^17^. However, that was a case-controlled study that involved a small number of cases (131 keratoconic eyes in total). Sarezky et al. conducted a large cohort study using claims data from a national insurance provider in the United States^13^ and reported that male sex, age between 20 and 40 years, African-American race, and a low level of education increased the risk of undergoing corneal transplantation. However, this study involved many older patients who might not have had active keratoconus since two-thirds of the participants were aged > 40 years. Considering that keratoconus mainly affects people aged < 40 years and corneal transplantation is only performed for active and severe forms of keratoconus, those aged > 40 years might have undergone corneal transplantation for other corneal pathologies. Therefore, the large proportion of older adults in the cohort may have affected the results and conclusions of the study. In our study, we prevented this by using data from the National Health Insurance database and only including patients between the ages of 10 and 40 years. Therefore, we believe that the present study provides more reliable, clinically-relevant information on the risk factors for corneal transplantation in patients with keratoconus.

We found that male sex was strongly associated with an increased risk of progression to corneal transplantation in patients with keratoconus. Numerous studies have reported that men have earlier disease onset and faster deterioration^23,24^. Recently, the role of sex hormones has been gaining support as an explanation of the more severe presentation of keratoconus in men. Sex hormones play a role in the maintenance of the structure and integrity of the human cornea, and hormone levels alter corneal thickness, curvature, and sensitivity at different points in the menstrual cycle^25^. However, the exact pathophysiological process is unknown and requires further investigation. We reported in our previous study that there was no difference in the incidence of keratoconus between men and women in South Korea^18^. However, in the present study, sex was associated with the rate of corneal transplantation. Therefore, we concluded that while there is no difference in the incidence of keratoconus between men and women, the severe forms may be more common in men. This is consistent with findings reported in the existing literature^23,24^.

Recent studies have noted that diabetes may have a protective effect on keratoconus^15,26^. The suggested pathophysiological basis for this effect is that the high blood glucose levels in diabetic patients cause glycosylation of the corneal stroma, leading to collagen cross-linking and stromal strengthening and thus the prevention of the development and progression of keratoconus^27,28^. However, in our study, diabetes was not found to be significantly associated with the prevention of keratoconus progression to corneal transplantation (P = 0.073), which we believe to be due to the small number of patients with diabetes and keratoconus included in this who underwent corneal transplantation. The proportion of patients with diabetes was small since active keratoconus usually occurs in patients who are aged < 40 years. Only one patient had diabetes and underwent corneal transplantation; therefore, it was difficult to identify statistically significant effects. Given the small HR for diabetes (0.164) in the present study, a longer follow-up period might have increased the number of patients included who underwent corneal transplantation and thus a clearer demonstration of the protective effect of diabetes mellitus may have been evident. Further studies are required to validate this finding.

Atopic dermatitis is a classic risk factor for the development and progression of keratoconus^29^. One of the most cited explanations is that pruritus in atopy leads to eye rubbing, which causes the cornea to wear out physically, leading to corneal thinning^30^. In addition to this hypothesis, studies have also shown that atopy and eye rubbing cause oxidative damage to the keratocytes, which leads to increased proteinase activity and keratocyte apoptosis^31,32^. Studies have also reported that human leukocyte antigens are associated with allergic conditions and that keratoconus candidate genes are linked to atopic dermatitis^29,33^. In our study, severe atopic dermatitis was linked to a greater risk of undergoing corneal transplantation, while a significant association failed to be demonstrated for overall atopic dermatitis. We suspect that atopic dermatitis in general would have resulted in non-specific and broad dermatologic findings in clinical practice, while only severe atopic dermatitis, which requires some medication for treatment, would have caused a certain degree of pruritus that would lead to an increased risk for corneal transplantation. In any case, our result confirmed that atopic dermatitis was associated with the progression of keratoconus, as previously established in the literature.

Our study also revealed that obstructive sleep apnea increased the risk of corneal transplantation in patients with keratoconus. Previously, Gupta et al.^34^ and Saidel et al.^35^ confirmed that a high proportion of patients with keratoconus have obstructive sleep apnea, which they suggested might be due to the presence of obesity, sleeping positions, and biomechanical alterations in related tissues (cornea and eyelid in keratoconus and airway in obstructive sleep apnea) in these patients. Sleep apnea was also reported to be associated with more severe keratoconus in a claims-based study conducted in the Unites States^15^, which is consistent with our results.

Several studies have also suggested a link between Down syndrome and keratoconus^1,14^. Some have suggested that it is difficult to teach patients with Down syndrome to not rub their eyes, which makes it more likely that these patients will exhibit uncontrolled eye rubbing, leading to the development and progression of keratoconus^15^. Additionally, for the same reason, keratoconus was reported to be highly prevalent among patients with intellectual disability^36^. In our study, both intellectual disability and Down syndrome significantly increased the risk of corneal transplantation in the univariate Cox regression; however, only intellectual disability was significantly associated with an increased risk of corneal transplantation in the multivariate analysis.

This study has several strengths. First, since the entire South Korean population is included in the database, this study included a large number of patients with keratoconus. This is the first large-scale longitudinal cohort study on the progression to corneal transplantation in patients with keratoconus performed in Asia, and unlike previous studies, only young patients with keratoconus were included. Additionally, our study revealed that the rate of progression to corneal transplantation in South Korea was lower than that in the United States^13^ and England^10^, and the risk factor trends were similar to those reported in previous studies despite the ethnic differences. These aspects indicate the novelty and relevance of our study.

However, our study also has some limitations. First, due to the nature of claims data, chart-level verification and tomographic confirmation of keratoconus were not possible; therefore, we could only analyze the data based on the diagnostic codes assigned by the practitioner. Nonetheless, the diagnoses were made by ophthalmologists, and since the diagnosis of keratoconus has improved due to advancements in corneal imaging techniques, we believe that the diagnostic codes used in our study were reliable. Second, we were unable to analyze the influence of collagen cross-linking since the procedure was not covered by insurance and could not be captured in the database. Third, we were unable to perform a per-eye-based analysis. Therefore, we could not determine whether the keratoconus was unilateral. Furthermore, for patients who underwent corneal transplantation twice, we could not determine whether they underwent bilateral primary surgery or unilateral repeated surgery. This prevented further in-depth analysis of the data. Fourth, the follow-up period was relatively short. We believe that if we were able to access keratoconus data over a longer period, it would have improved the findings of our study. However, the HIRA Deliberative Committee only permitted access to the data from January 2009 to June 2015, which is an intrinsic limitation of our study. Finally, only 124 patients underwent corneal transplantation, which might not have been sufficient to allow for the identification of significant risk factors for corneal transplantation. If we could have collected data over a longer time period, additional patients who underwent corneal transplantation might have been included, potentially leading to more meaningful results. This limitation should be addressed in future studies.

In conclusion, our nationwide population-based cohort study of keratoconus in South Korea provides data on the incidence of and several risk factors for the progression to corneal transplantation in this patient population. Patients with keratoconus in South Korea have a relatively low rate of corneal transplantation compared to other countries. Male sex and the presence of atopic dermatitis, obstructive sleep apnea, and intellectual disability increase the risk of corneal transplantation in patients with keratoconus. This study may provide valuable insight regarding the disease burden of keratoconus and improve our understanding of the pathophysiology of this disease.

## Supplementary Information

Supplementary Table S1.

## Data Availability

The data that support the findings of this study are available from the Health Insurance Review and Assessment Service of South Korea; however, since these data were used under license for the current study, they are not publicly available. Data will be made available from the authors upon reasonable request and with permission of the Health Insurance Review and Assessment Service of South Korea.
