# Supplementary material for: Corneal transplantation for keratoconus in South Korea
Source: Sci Rep. 2021 Jun 15;11:12580. doi: 10.1038/s41598-021-92133-y (PMC8206092; doi:10.1038/s41598-021-92133-y)
Supplement: Supplementary file 1 — Supplementary Table S1. [file 41598_2021_92133_MOESM1_ESM.pdf]

Corneal Transplantation for Keratoconus in South Korea

Sungsoon Hwang, MD[1,2,*], Tae-Young Chung, MD, PhD[1,*], Jisang Han, MD, PhD[3], Kyunga Kim, PhD[4,5], Dong Hui Lim, MD, PhD[1,2]

[1]Department of Ophthalmology, Samsung Medical Center, Sungkyunkwan University School of Medicine, Seoul, Republic of Korea

[2]Department of Clinical Research Design & Evaluation, Samsung Advanced Institute for Health Sciences and Technology, Sungkyunkwan University, Seoul, Republic of Korea

[3]Department of Ophthalmology, Kangbuk Samsung Hospital, Sungkyunkwan University School of Medicine, Seoul, Republic of Korea

[4]Statistics and Data Center, Research Institute for Future Medicine, Samsung Medical Center, Seoul, Republic of Korea.

[5]Department of Digital Health, Samsung Advanced Institute for Health Sciences and Technology, Sungkyunkwan University, Seoul, Republic of Korea

*These authors contributed to this article equally as the first authors.

Correspondence

Dong Hui Lim, MD, PhD

Department of Ophthalmology, Samsung Medical Center, Sungkyunkwan University School of Medicine, #81 Irwon-ro, Gangnam-gu, Seoul 06351, Korea

Tel: +82-2-3410-3548, Fax: +82-2-3410-0074

E-mail: ldhlse@gmail.com

Supplementary Table S1. The diagnostic and medication codes used to define pre-existing diseases

**Procedure**	**Surgical code**
Anterior lamellar keratoplasty	S5371
Penetrating keratoplasty	S5372
**Diseases**	**Diagnostic code (Korean Standard Classification of Diseases, 7th)**
Keratoconus	H18.6
Hypertension	I10.*, I11.*, I12.*, I13.*, I15.*
Diabetes mellitus	E10.*, E11.*, E13.*, E14.*
Dyslipidemia	E78.*
Atopic dermatitis	L20.*
Asthma	J45.*
Allergic rhinitis	J30.*
Collagen vascular disease	M30.*, M31.*, M32.*, M33.*, M34.*, M35.*, M36.*
Aortic aneurysm	I71.*
Mitral valve prolapse	I34.1
Obstructive sleep apnea	G47.3
Intellectual disability	F70.*, F71.*, F72.*, H73.*, F78.*, F79.*
Down syndrome	Q90.*
Turner syndrome	Q96.*
Marfan syndrome	Q87.4
Ehlers–Danlos syndrome	Q79.6
Osteogenesis imperfecta	Q78.0
**Medication**	**Medication code**
Systemic steroid	116401ATB, 116501ATB, 296900ATB, 116502BIJ, 141901ATB, 141903ATB, 141904ATB, 142001BIJ, 142201BIJ, 142202BIJ, 170901ATB, 170905ATB, 170906ATB, 171201BIJ, 171202BIJ, 171204ATB, 193301ATB, 193302ATB, 193303ATB, 193501BIJ, 193502BIJ, 193601BIJ, 193602BIJ, 193603BIJ, 193604BIJ, 193605BIJ, 217001ATB, 217003ASY, 217004ASY, 217104BIJ, 217301BIJ, 217302BIJ, 243201ATB, 243202ATB, 243203ATB, 243301BIJ, 243303BIJ, 243305BIJ, 316100BIJ
Cyclosporine	139201ACS, 139201ALQ, 139204ACS, 194701ALQ, 194701ACS, 194702ACS, 194703ACS, 139202BIJ
Azathioprine	139202BIJ, 112401ATB
Mycophenolate mofetil	197801ACH, 197802ATB
Methotrexate	192101ATB, 192107ATB, 192101BIJ, 192102BIJ, 192103BIJ, 192104BIJ, 192105BIJ, 192107BIJ, 192108BIJ, 192109BIJ, 192110BIJ, 192111BIJ, 192112BIJ
Rituximab	422601BIJ, 422602BIJ
Omalizumab	572901BIJ

Immunoglobulin	169901BIJ, 169902BIJ, 169903BIJ, 169904BIJ, 169905BIJ
Interferon-**γ**	175901BIJ
